# Trust in addiction treatment services among people who use drugs in Vietnam

**DOI:** 10.1016/j.josat.2026.209898

**Published:** 2026-01-16

**Authors:** Li Li, Li-Jung Liang, Thang Hong Pham, Ha Thi Thanh Nguyen, Tuan Anh Nguyen

**Affiliations:** a Department of Psychiatry and Biobehavioral Sciences, David Geffen School of Medicine, University of California, Los Angeles, 760 Westwood Plaza, Los Angeles, CA, 90024, USA; b Division of General Internal Medicine and Health Services Research, David Geffen School of Medicine, University of California, Los Angeles, 1100 Glendon, Los Angeles, CA, 90024, USA; c National Institute of Hygiene and Epidemiology, 1 Yersin Street, Hanoi, 100000, Viet Nam

**Keywords:** Trust in addiction services, Treatment stigma, People who use drugs, Vietnam

## Abstract

**Background:**

Although trust in addiction treatment providers and trust in treatment services are related yet distinct constructs, the latter remains underexplored. In Vietnam, where addiction treatment is transitioning from punitive models to community-based harm reduction, research on trust in treatment systems is particularly limited.

**Methods:**

Baseline data were drawn from an ongoing randomized controlled trial conducted across three provinces in Vietnam, involving 690 people who use drugs (PWUD). Data were collected through face-to-face, computer-assisted interviews using validated and adapted measures in addiction treatment services, depressive symptoms, perceived care support, and internalized stigma related to methadone maintenance treatment (MMT). A linear mixed-effects regression model with commune-level random-effects was used to assess associations between trust in addiction treatment services and the measures of interest, including demographic characteristics.

**Results:**

Higher levels of internalized MMT stigma and depressive symptoms were both significantly associated with a decreased trust in addiction treatment services in the adjusted analysis. Demographic characteristics and current MMT status were not significantly associated with trust. Although trust in addition services was associated with perceived care support in bivariate analysis, this relationship was no longer significant after adjusting for other factors.

**Conclusion:**

These findings suggest trust in addiction treatment could be understood not only as a function of provider-patient relationships but also in broader institutional and systemic contexts. Addressing treatment-related stigma and integrating mental health services in addiction services may be promising strategies to improve PWUD's trust in addiction services.

## 1. Introduction

Although patients' trust in addiction treatment providers and in treatment services are interrelated, they are conceptually distinct. As trust in providers is more cultivated through observed providers' empathy, nonjudgmental attitudes, and responsiveness to patient concern, trust in treatment services refers to patients' confidence in the efficacy of service approaches, including medications and treatment models (Gilson, 2003; Hall et al., 2001; Lago et al., 2017). However, the existing literature on trust in services has predominantly focused on provider-related trust (Hall et al., 2002; LaVeist et al., 2009; Makarenko et al., 2016; Ozawa & Sripad, 2013). This presents a critical research gap, as mistrust in treatment may impede engagement and hinder long-term outcome, even when provider trust is present.

Previous studies have explored several factors influencing people who use drugs (PWUD) and their trust in treatment services. Some research has shown that imposed identities on PWUD and negative care experiences significantly shape trust in healthcare and addiction treatment (Biancarelli et al., 2019; Cockroft et al., 2019; Harris & McElrath, 2012; Treloar et al., 2016; van Boekel et al., 2013). Other studies reported that strong social support has been positively correlated with increased trust in treatment and greater engagement in care (Dobkin et al., 2002; Tracy & Wallace, 2016). These studies underscore the multidimensional nature of trust in treatment and highlight the need for further investigation.

Trust in addiction services remains an underexplored concept in low- and middle-income countries (LMICs), where health systems are often under-resourced and sociocultural attitudes toward treatment can be complex. In recent years, Vietnam has made noteworthy strides in transitioning from a punitive, criminal justice-based approach to a more health-oriented treatment model (Nong et al., 2022). As the country continues to expand and decentralize its addiction treatment infrastructure toward community-based models (Nguyen et al., 2023), a deeper understanding of the role and dynamics of trust in shaping treatment engagement and experience is critical. This study seeks to contribute to that understanding.

## 2. Methods

### 2.1. Participants

This study used the data collected at the baseline of an ongoing randomized controlled intervention trial in three provinces (Ninh Binh, Da Nang and Can Tho) in Vietnam (Li et al., 2023). From May to July 2024, assessments were collected from 58 communities of the three provinces. The participants in this study were Vietnamese people who use drugs (PWUD) who resided in the catchment area of the 58 communities. The inclusion criteria were (1) aged 18 and above, (2) having a history of opioid use, (3) having disclosed opioid-using status to at least one of his/her family members and is willing to invite this family member to our study, (4) currently residing in the selected communes, and (5) providing informed consent. At baseline, a total of 690 people with a history of drug use were recruited, assessed, and included in this study.

### 2.2. Data collection

Before any data collection, written informed consent was obtained from all participants. Trained interviewers administered the assessment in a one-on-one, face-to-face format using a computer-assisted personal interview (CAPI) method. The interviewers asked participants assessment questions and keyed in their answers directly on laptop computers. All questions were asked in Vietnamese, and participants spent approximately 45 min completing the assessment. Each participant received 230,000 VND (approximately $10.00 USD) as compensation for their time and effort. The refusal rate of participation was approximately 15%.

### 2.3. Measures

*Trust in Addiction Treatment* was measured with six questions adapted from the LaVeist and colleagues study the validity and reliability of the Medical Mistrust Index (LaVeist et al., 2009). We revised the six statements and replaced “health care organization” with “drug use treatment services”. We defined drug use treatment services as evidence-based, outpatient methadone maintenance treatment (MMT) programs, which were the only officially accessible treatment option available at the time of data collection. To ensure clarity for participants, interviewers were trained to read the following: “There are some drug use treatments such as MMT services available now. Please tell us how much you agree or disagree with the following statements.” The statements included (1) I need to be cautious when dealing with available drug use treatment services, (2) I think patients have sometimes been deceived or misled by drug use treatment services, (3) I think drug use treatment services sometime cover the mistakes that they make, (4) I think these drug use treatment services have sometimes done harmful experiments on patients without their knowledge, (5) I think these drug use treatment services don't keep patients' information totally private, and (6) Sometimes I wonder if the drug use service providers really know what they are doing.” Responses for each item was measured on a 5-point scale with 1 = strongly disagree to 5 = strongly agree. All items were reverse-coded, and a composite score was computed, with a higher score indicated a higher level of trust in addiction treatment services (Cronbach's alpha = 0.77).

*Depressive symptoms* were measured using the short version of the Zung Self-Rating Depression Scale (Zung, 1965). We used six items adapted from the original 20-item questionnaire. Participants were asked how often they felt each of the six situations, including (1) You feel downhearted and blue, (2) You have trouble sleeping at night, (3) You notice that you are losing weight, (4) You get tired for no reason, (5) You are restless and cannot keep still, and (6) You are more irritable than usual. Response categories were from (1) “a little of the time” to (5) “all the time.” The overall scale was the sum of the individual items, as a higher score indicated a higher level of depressive symptoms (Cronbach's alpha = 0.76).

Perceived s*upport for care* was assessed by a scale of 9 items asking how often that each type of support was available if needed. Responses were recorded on a 5-point scale ranging from (1)“none of the time” to (5) “all of the time.” This was following the same format used in a social support scale developed by Sherbourne and Stewart (1991). For this study, we included nine care related support areas: (1) to take you to the doctor if you are sick, (2) to give you information to help you find medical/health services, (3) to help you to understand your health condition, (4) to give you advice about available service and treatment, (5) to provide financial support for your seeking care, (6) to remind you to take medication, (7) to take you to testing sites, (8) to remind you to refill your medication, and (9) to provide financial support for your staying in treatment. Participants were asked how often each kind of support was available it they need it. Scores were the sum of ratings from all the nine areas, with a higher score indicating a better level of support for care (Cronbach's alpha = 0.94).

*Internalized MMT stigma* was assessed with a 6-item scale originally developed by Smith et al. (2020). Participants were asked how much they agree or disagree with these statements: (1) Receiving methadone makes me feel like I am a bad person, (2) I feel I am not as good as others because I receive methadone, (3) I feel ashamed of my methadone treatment, (4) I think less of myself because I receive methadone, (5) Receiving methadone makes me feel unclean, and (6) Being on methadone treatment makes me feel like I am still a drug user. Response categories ranged from 1 = strongly disagree to 5 = strongly agree. All scores were summed, and a higher summary score indicated a higher level of internalized MMT stigma (Cronbach's alpha = 0.96).

Participant demographic characteristics such as age, gender, marital status, and education were collected. Current MMT status, measured by the single question if currently in MMT treatment program, was also included in the study.

### 2.4. Data analysis

Descriptive statistics and frequencies summarized participants' demographic characteristics, including gender, age, marital status, education level, and current methadone maintenance treatment (MMT) status. Pearson correlations were used to explore bivariate relationships among measures of interest: trust in addiction treatment services, depressive symptoms, perceived care support, and internalized stigma.

To assess the relationship between trust in addiction treatment services and measures of interest, we employed a linear mixed-effects regression model with commune-level random-effects to account for repeated observations within communes. Demographic variables were included as covariates. Estimated regression coefficients, standard errors (SEs), and their corresponding significance levels were reported. All statistical analyses were performed using SAS software, version 9.4 for Windows (SAS Institute, Cary, NC).

## 3. Results

Of the 690 PWUD participants, the majority (96.8%) were male. The average age was 42 years; nearly half (49%) were between 36 and 45 years old, while 21% were aged 18 to 25. Most participants (58%) were married or living with a partner at the time of assessment. Regarding education level, 59% had completed at least 9 years of education, whereas 14% had received 5 or fewer years. Nearly 79% of participants reported being enrolled in a methadone maintenance treatment (MMT) program at the time of the survey. Among participants not currently enrolled in MMT, 37 reported previous discontinuation, most commonly due to a desire to stop methadone or conflicts between MMT schedules and daily routines. No significant demographic differences were observed between participants currently enrolled and those not enrolled.

Descriptive statistics for the measures of interest are also presented in Table 1. Bivariate correlations indicated that trust in addiction treatment services was negatively associated with depressive symptoms (correlation = −0.23, *p* < 0.001) and internalized MMT stigma (−0.46, p < 0.001), but positively associated with perceived care support (0.13, p < 0.001).

Results from the linear mixed-effects regression are presented in Table 2. Consistent with the bivariate relationships, internalized MMT stigma (Estimate = −0.284, SE = 0.031, *p* < 0.0001) and depressive symptoms (Estimate = −0.161, SE = 0.043, *p* = 0.0002) were both negatively associated with trust in addiction treatment services. No significant associations were observed between trust in addiction services and participants' demographic characteristics, current MMT status, or perceived care support.

## 4. Discussion

In this study, we examined the trust that PWUD place in addiction treatment services, drawing on the conceptual framework of trust articulated by Hall et al. (2001). In this model, trust is directed not only toward individual service providers but also toward institutions and broader medical systems. Previous studies have indicated that trust is tied to vulnerability and that sustaining trust requires a balance between risk and protective mechanisms (Hall et al., 2001; Mechanic & Meyer, 2000). For individuals marginalized by dominant social structures, such as PWUD, trust is often associated with greater perceived risk, and negative experiences within general health care settings may diminish PWUD's willingness to engage with or adhere to addiction treatment services. It was reported that many PWUD patients felt they were viewed as inherently untrustworthy due to pervasive stereotypes about drug use (Buchman et al., 2016; Harris et al., 2013). Over time, these experiences and observations may shape their expectations about whether they will be perceived as trustworthy in other health care encounters. Therefore, trust in addiction treatment must be understood as a dynamic process that extends beyond interpersonal interactions to encompass institutional and systemic dimensions.

It is worth noting that we found no significant association between trust in addiction services and current MMT status in our study sample. The primary focus of this study is to examine trust as a broader construct and its correlates, rather than to predict treatment enrollment. This finding, however, highlights the need to consider other determinants of treatment engagement and how they may intersect with trust in services. Factors such as prior treatment discontinuation and history of injection drug use may also influence trust and warrant further investigation in future studies. Furthermore, the age distribution of our sample suggests that many participants in this study came of age during the period when drug use in Vietnam was legally framed as a crime rather than a health condition. The legacy of punitive approaches and social stigma from earlier decades may continue to shape perceptions of care. Notably, our multivariate models showed no significant association between age and trust in treatment services. Future research should explore multilevel dynamics to understand how trust is built and sustained in different contexts.

Our study indicates that internalized MMT stigma is a significant factor associated with PWUD's trust in addiction treatment services. While much of the existing literature has examined stigmatizing attitudes among service providers toward PWUD, our study shifts the focus to stigma internalized by PWUD themselves, specifically related to methadone treatment, and its implications for trust in addiction services more broadly. Paquette et al. (2018) found that some individuals perceived greater public stigma associated with methadone treatment than with injecting drugs, due to the public perception of methadone as equivalent to illicit drug use. Harris and McElrath (2012) highlighted the interplay of social control and institutional stigma in patients' accounts of MMT experiences, suggesting that such linkages may reinforce “drug addict” identities in public contexts. Treloar et al. (2016) further showed how PWUD's trust in service programs is shaped by their perceived identity as service users versus drug users, arguing that stigmatized identities can undermine trust in care. These theoretical frameworks inform our interpretation of the observed relationship between internalized MMT stigma and diminished trust in addiction services. In Vietnam, where drug use was historically criminalized, building trust in treatment services remains a critical challenge. Although policy reforms have increasingly emphasized a health-oriented approach, stigma toward methadone treatment could continue to undermine its acceptance. This stigma persists even though methadone treatment is internationally recognized as an evidence-based intervention grounded in harm reduction principles and intended to treat drug use as a chronic health condition. Addressing stigma surrounding methadone treatment may therefore represent an essential first step toward building and strengthening trust in addiction services.

In this study, we found that trust in addiction treatment was negatively associated with depressive symptoms. This finding is consistent to previous research indicating that patients' mental health was closely intertwined with their trust in treatment. Greater psychological comfort may enhance perceptions of treatment services, while trust in those services may, in turn, contribute to psychological well-being and treatment satisfaction (Hall et al., 2002; Ozawa & Sripad, 2013). Long-term adherence and successful treatment outcomes depend heavily on sustained motivation, which can be particularly challenging for PWUD with limited resources. As such, routine screening for depressive symptoms and the provision of accessible mental health services may help foster trust and engagement in addiction treatment.

These findings should be interpreted within the context of several limitations. First, the cross-sectional design restricts conclusions to associative relationships and precludes causal inferences. Second, our sample was largely composed of individuals currently enrolled in MMT and who had disclosed their drug use to family, which may limit generalizability to PWUD not engaged in treatment or with negative experience that could also affect trust and stigma. Third, our findings should be understood within the specific policy and service context of Vietnam, where treatment services remain primarily limited to methadone, and may have limited generalizability to settings that provide broader, multi-modality treatment options through more diverse organizational models. Fourth, our study did not include measures of patient and treatment provider trust, which restricted our ability to examine whether trust in providers and trust in the treatment system are empirically distinct or overlapping constructs. Nevertheless, this study, leveraging a relatively large sample size, provides valuable insights into the trust that PWUD place in addiction services and its association with treatment-related stigma and individual factors such mental health. Importantly, these factors are potentially modifiable, presenting opportunities to develop targeted interventions responsive to the special needs of this population and to improve treatment engagement and outcomes.

### 4.1. Conclusion and implications

Our study findings that internalized MMT stigma and mental health challenges are key barriers to trust in addiction treatment among PWUD have important implications for addiction treatment services in Vietnam. As addiction treatment services continue to expand and integrate into community settings, a critical challenge for program designers and policymakers is how to enhance the trust of PWUD in the service system. Unlike interpersonal trust, which is primarily built through direct interactions with providers, program-level trust is often shaped by patients' broader confidence that treatment services will act in their best interests. This form of trust is more abstract and influenced by general perceptions and knowledge of available services. Efforts to enhance trust could address these broader perceptions through targeted social marketing and evidence-based interventions to build a trustworthy environment, thereby supporting sustained engagement and improved treatment outcomes.

In addition to addressing general perceptions and expanding clinical services such as mental health care, structural and policy-level reforms are needed to reduce systemic barriers that constrain addiction treatment in Vietnam. Existing institutional frameworks, characterized by limited treatment modalities and rigid program structures, continue to restrict access to care. Broadening treatment options and integrating addiction services with other health and prevention programs may help reduce stigma and strengthen accessibility and trust in treatment systems.

## Figures and Tables

**Table 1:** Sample characteristics and measures of interest (*N* = 690).

	% (N) or mean (SD)
Gender	
Male	96.8 (668)
Female	3.2 (22)
Age, mean age (SD)	42.2 (8.8)
18 to 35	20.9 (144)
36 to 45	48.9 (338)
46 to 55	23.9 (165)
56 and above	6.2 (43)
Marital status	
Single	25.5 (176)
Married/living with partner	57.8 (399)
Divorced/separated/widowed	16.7 (115)
Years of education, mean (SD)	8.78 (3.33)
5 years or fewer	14.3 (99)
6–8 years	27.0 (186)
9–11 years	32.9 (227)
12 years or more	25.8 (178)
Currently in MMT	77.8 (537)
	Mean (SD)
Trust in addiction treatment serves	25.2 (4.3)
Depressive symptoms	9.6 (3.6)
Support for care	31.5 (8.6)
Internalized MMT stigma	12.5 (5.0)

SD: Standard deviation.

**Table 2:** Summary of linear mixed-effects regression results (N = 690).

	Estimate	SE	P
Male (REF = Female)	−1.065	0.819	0.1938
Age (REF = Older than 55)			
18–35	−0.232	0.716	0.7458
36–45	0.352	0.646	0.5862
46–55	0.379	0.661	0.5670
Marriage (REF = Divorced/separated/widowed)			
Single	−0.327	0.452	0.4694
Married or living with your partner	0.064	0.398	0.8719
Years of education (REF = 12 or more)			
5 or less	−0.205	0.490	0.6752
6 to 8	−0.623	0.416	0.1350
9 to 11	−0.619	0.355	0.0818
Currently in MMT	−0.272	0.581	0.6394
Care Support	0.009	0.018	0.6288
Depressive Symptoms	−0.161	0.043	0.0002
Internalized MMT stigma	−0.284	0.031	<0.0001

SE: Standard error.
